# Book review of: "Clinical aspects of electroporation" by Stephen T Kee, Julie Gehl, Edward W Lee

**DOI:** 10.1186/1475-925X-10-89

**Published:** 2011-10-07

**Authors:** Enrico Pierluigi Spugnini

**Affiliations:** 1 SAFU Department Regina Elena Cancer Institute, Via delle Messi d' Oro 156, 0158 Rome, Italy

**Keywords:** electroporation, electrochemotherapy, irreversible electroporation

## Abstract

This article is a review of the book: Clinical aspects of electroporation, by Stephen T. Kee, Julie Gehl, Edward W Lee, which is published by Springer Press. Basic information that should be helpful in deciding whether to read the book and whether to use it as a reference book is presented. This includes an introduction, a description of all the sections of the book, and a comparison with recently published books on the topic.

## Book Information

KeeStephen TGehlJulieLeeEdward WClinical aspects of electroporation2011New York: SpringerPrice: € 128.50. ISBN 978-1-4419-8362-6 # 256 pages, Binding: Hardback

## Introduction

Electroporation is a technique to drive molecules in intracellular targets that is gaining momentum in basic science and clinical practice. This book is focused on providing the reader with a concise yet thorough summary of the recent breakthroughs in the field of electroporation. It comprises 21 chapters divided in four parts: introduction, electrochemotherapy, gene electrotransfer and irreversible electroporation. The book is overall well structured and easy to follow. The book can be used in part as an excellent reference work for biomedical engineers, scientists, and clinicians.

## Systemic description of the opera

### Part I. Introduction

The introduction provides the reader with a comprehensive review of the physics and physiology of electroporation. In particular, there is a clear description of the phenomena that occur at the cellular level after the application of electric pulses. The transient and permanent effects of different types of electrical stimulations are discussed in depth and described and illustrated in a manner to be clearly understood by readers who are not familiar with the topic. The difference between reversible electroporation (transiently increased cell permeability) and irreversible electroporation (non-reversible cell permeability resulting in altered cross-membrane ion flow and ultimately in cell death) are clearly described. The mathematical and physical aspects of electroporation are described with a practical approach in order to be helpful for the basic scientist and the clinical investigator.

The chapter on different equipments at the end of the introduction is more similar to a company catalogue than a book chapter. Perhaps a broader discussion of the advantages and disadvantages of the different waveforms and electrical protocols would have been more useful.

### Part II. Electrochemotherapy

The second part of the book is focused on electrochemotherapy and in the first two chapters the basics of this technique are described at the cellular level and at the vascular level. More precisely, the increased captation of chemotherapy drugs and the electroporation-induced vascular disruption are explained very well.

The clinical experience on cancer patients is limited to two chapters, the first one summarizes the experience of the ESOPE Group on small tumors (especially melanoma). This chapter is well structured with a section devoted to guide clinicians on techniques to treat dermatological malignancies. The photographic support is more than adequate. Unfortunately the chapter does not report the experience of other groups outside the ESOPE study. Considering the small number of groups working worldwide on this therapy, this is a significant limitation for the reader.

The second chapter is devoted to the description of the clinical approach to large malignancies, especially the palliation of large cutaneous metastases. Again, the chapter is well-written and exhaustive, with a good photographic support and a good algorithm to guide the clinicians. This section could have been potentially strengthened by adding a small report on the experience in veterinary patients, where large tumors have been successfully treated over the past decades.

The last three chapters, despite being well written, are somewhat disappointing because they focus on electroporation in the bone, brain and organ lumens but the discussion of the subject is limited to the experimental results obtained in laboratory animals and one dog with a spontaneous neoplasm.

### Part III. Gene electrotransfer

The third part is focused on gene electrotransfer and thoroughly describes the different strategies for using this technique for curative and prophylactic purposes (gene therapy and DNA vaccine). The different chapters point out the many challenges posed by gene delivery in different biological targets such as tumors, skin, muscle and lungs. While most of the presented data are obtained from laboratory animals, the author strive to translate their experience into a patient-oriented setting, providing several insights potentially useful to clinicians willing to start clinical trials on this therapy.

### Part IV. Electroporation

The fourth and last part is focused on a recently-developed electroporation-based strategy: the direct tumor ablation by using irreversible electroporation (IRE). The three chapters of this section are thorough, well written and show a sequential construction. The first chapter describes the basics of irreversible electroporation and the first preclinical experience on rabbit and swine liver; the second chapter reports the results of IRE in a rabbit model of head and neck cancer, and finally the third chapter is focused on the translation of the above results to human patients with liver metastases. The imaging support of the chapters is very comprehensive and allows an easy grasp of the fundamentals of this technique, helping the reader to understand the state of the art of IRE.

## Comparison with the current literature

There is currently little available for readers except for few other books such as:

1) Irreversible Electroporation By: Boris Rubinsky; New York: Springer; 2010. Price: $ 169. ISBN 978-3-642-05419-8 # 328 pages, Binding: Hardcover, 13 chapters. In this book there is the deepest analysis of irreversible electroporation from basic studies to clinical applications, the authors are well known opinion leaders in the field. Considering that the book is exclusively focused on irreversible electroporation, it has an in depth analysis that cannot be reached by books that deal with a broader vision of electroporation. On the other hand, this book lacks completely a discussion on all the other aspects of electroporation.

2) Electroporation in Laboratory and Clinical Investigations By: Enrico Spugnini and Alfonso Baldi; New York: Nova Science; 2011. Price $ 145. ISBN: 978-1-61668-327-6 ISBN: 978-1-61668-383-2 Binding: Hardcover, 18 Chapters. This book offers a comprehensive and up to date overview on electroporation in mathematic modeling, bioengineering, molecular biology, plant biology, pathology, veterinary and human oncology. This book provides the reader with an in depth analysis of the mathematical and physical basis of electroporation, describes the different types of electroporation currently adopted and has a very good clinical section, strengthened by two chapters of electroporation in veterinary oncology that give the book an edge.

3) Advanced electroporation techniques in biology and medicine By: Pakhomov Andrei G.; Miklavcic Damijan; Markov Marko S. Boca Raton: Florida; CRC Press; 2010. Price € 112. ISBN 1439819068 Binding: Hardcove, 29 Chapters. This book summarizes most recent experimental findings and theories related to permeabilization of biomembranes by pulsed electric fields. It focuses on biophysical mechanisms of electroporation and applications of this phenomenon in biomedical research and medicine. It provides a broad examination of the discoveries and clinical orientations in this field.

## Final considerations on "Clinical aspects of electroporation"

Despite some limitations, there is a clear intent to provide as much information as possible to researchers interested in adopting electroporation techniques. Unfortunately, the title is misleading, since the clinical part is somewhat limited. As a result, this book will be more helpful to basic scientists than clinicians; however it is definitely a book worth having as a reference.

## Competing interests

The author declares that they have no competing interests.

## Authors' contributions

EPS is the sole author

## Author information

DVM, Ph D, Diplomate ACVIM (Oncology), Diplomate ECVIM-CA (Oncology)
